# Diagnostic Approach to Reflux in 2007

**DOI:** 10.1155/2008/367320

**Published:** 2008-08-28

**Authors:** Sumit Dave, Antoine E. Khoury

**Affiliations:** ^1^Division of Urology, Children's Hospital, London Health Sciences Centre, London, Ontario, Canada N6A 5W9; ^2^Division of Urology, UC Irvine Healthcare, UC Irvine, Orange, CA 92868, USA

## Abstract

There is ongoing controversy regarding the association between vesicoureteric reflux (VUR), recurrent urinary tract infections (UTI), and renal damage. Despite this, routine work up for VUR is still recommended after febrile UTI in most children. The present article reviews the indications and imaging modalities available for VUR diagnosis. Alternative newer techniques like MR cystography and voiding urosonography are discussed. The increasing evidence of the role of DMSA scans in managing children with VUR is highlighted.

## 1. INTRODUCTION

The goals of an imaging procedure in general are to confirm the diagnosis suspected with a high degree of sensitivity and specificity, to aid treatment and allow prognostication. On the other hand, it is obligatory for the treating physician to analyze the risks and benefits of the diagnostic procedure and understand the natural history of the disease in question to establish whether or not the diagnosis and treatment of a condition would alter long term outcome or impact management decisions.

The diagnosis of vesicoureteric reflux (VUR) is a relatively straightforward and well-established procedure. However, the underlying rationale for identifying VUR to prevent recurrent pyelonephritis (PN) and long-term renal damage has been vigorously questioned in the recent literature [1–4]. Coupled with this, there has been the increasing awareness of the risks of radiation exposure and the realization that VUR investigation is an invasive procedure and definitely an unpleasant experience. Therefore, it is imperative for the pediatric urologist and nephrologist to reevaluate the indications and goals for imaging for VUR, redefine the modalities used, and establish guidelines for followup.

The current article reviews the available modalities for evaluating VUR, suggests protocols for investigating children with suspected VUR, and presents the recent evidence justifying these recommendations.

## 2. IMAGING MODALITIES FOR DIAGNOSIS OF VUR

### 2.1. Voiding cystourethrogram (VCUG) or radionuclide cystogram (RNC)

An ideal test for VUR detection would be one involving no radiation, no bladder catheterization, no sedation, low cost, high sensitivity, and one which provides complete anatomical details. The traditional method for diagnosing VUR is the fluoroscopic voiding cystourethrogram (VCUG). Currently, radionuclide cystography (RNC) remains the primary alternative to a VCUG in evaluating VUR. The objection to performing a VCUG is related to the high radiation exposure of a traditional VCUG which is believed to be about 100 times that of a RNC. However, with the judicious use of digital and pulsed fluoroscopy with meticulous image coning, the radiation exposure from a VCUG has been significantly reduced [5]. Despite these measures, the average ovarian radiation dose was shown to be about 10 times greater in a VCUG when compared with an RNC [6]. The proponents of RNC argue that this effectively reduces the sensitivity of the VCUG in identifying VUR as snapshots of the bladder filling and voiding are taken rather than continuous imaging. In an editorial comment, Benson stated that “the radiation dose given during 3 minutes of fluoroscopy time approximates that of two pelvic spiral CT scans with contrast, 1.5 conventional abdominal CT scans without contrast, 3 DMSA scans, 60 abdominal plain films, 600 radionuclide VCUG’s, or 10 years of background radiation” [7].

Several studies have compared the sensitivity of VCUG and RNC and concluded that RNC is at least as sensitive as or more than a VCUG for detecting VUR [8–10]. Sükan et al. observed no significant difference between the 2 modalities but noted that RNC offered a higher sensitivity in the younger age group [8]. Moreover, despite not reaching statistical significance, in children with a positive dimercapto-succinic acid (DMSA) scan, RNC identified VUR in a higher percentage of children as opposed to the VCUG. This is a relevant finding in the current era where a positive DMSA scan is believed to be an important finding in children presenting with PN (as discussed later). An insignificant grade 1 VUR may be missed on an RNC but the continuous imaging during filling and voiding allows greater detection of VUR, which is an intermittent phenomenon. For both tests, it is well established that a cyclic study should be performed to increase the detection of VUR [11]. The definite advantage a VCUG has over an RNC lies in the anatomical information obtained by the former especially in evaluating the urethra in males. Moreover, the International Reflux grading system is based on the VCUG and since most studies on VUR have used this grading system, treating physicians feel more comfortable in knowing this information. However, again as a result of change in treatment philosophy, it can be argued that a broader classification of VUR into dilating (≥Grade 3) and nondilating varieties is possible on an RNC, making this an insignificant issue when deciding on the study to obtain. Therefore, currently VCUG remains the standard when initially evaluating a child with suspected VUR. For all subsequent studies, the RNC is the preferred modality. Medina et al. performed a cost analysis of VUR imaging using VCUG and RNC, highlighting another potential reason for preferring the RNC for followup imaging [12]. The study showed that the direct costs of performing a VCUG was 1.74 times higher than an RNC (*P* < .001).

### 2.2. Indirect RNC

The indirect RNC offers the possibility of detecting VUR without bladder catheterization and presumably in a more physiological setting with natural bladder filling. The additional advantage is the ability to assess upper tract differential function and drainage with the injected radioisotope. Although a few reports have shown a comparable degree of sensitivity between the direct and indirect RNC, the consensus is that due to an inability to study the filling phase with an indirect RNC there is a considerable false negative rate with indirect RNC [13–15]. There may be a role of the indirect RNC as a followup study in children who are toilet trained [14].

### 2.3. Voiding urosonography (VUS)

The sonographic evaluation of VUR following intravesical instillation of US contrast agent has gradually popularized VUS over the last decade. In a comprehensive review of VUS when compared with VCUG, Darge showed that in 1338 patients with 2893 refluxing units, VUS showed a diagnostic accuracy of 78%–96% [16]. The overall agreement between the 2 studies was 91% and in 9% of renal units VUR was detected only on VUS making it the more sensitive study. Another potential advantage of the VUS as compared to RNC is the ability to grade VUR similar to the International Reflux grading system with a 75% concordance shown between the VUS and VCUG grading. The discordant findings are primarily because of a significant number of grade-1 VUR on VCUG being grade 2 or higher on VUS. The absence of radiation, the ability to evaluate the upper tracts simultaneously and its higher sensitivity make it an attractive tool for evaluating VUR. The potential drawbacks include inadequate evaluation of bladder and urethral morphology, higher costs of contrast agent, longer exam time, and its operator dependence. Transperineal US of the urethra as part of the VUS has been used reliably in a few studies to date but further evaluation of this technique is necessary before VUS can replace the VCUG as the first examination for VUR especially in boys [17]. At present, VUS has a role in followup examinations for VUR, for screening siblings and possibly as the first examination for VUR in girls.

### 2.4. MR voiding cystography (MRVCUG)

MR cystography involves intravesical administration of gadolinium with imaging using MR during filling and voiding. The relative benefits of the procedure are that it can evaluate VUR without ionizing radiation and additionally give important information about renal-acquired cortical defects and differentiate acquired cortical defects from congenital dysplasia. It must be borne in mind that dysplasia and scarring are different entities diagnosed on histopathological examination. In this paper we refer to them as congenital and acquired cortical defects. The potential drawbacks include a lower sensitivity as compared to VCUG, higher costs, and the need for sedation or anesthesia to perform the study. Takazakura et al. showed 90% sensitivity and a 96% specificity of MRVCUG and all children with grade 3 or more VUR were identified using this modality [18]. Lee et al. further correlated the MRVCUG findings with a DMSA SPECT scan to demonstrate the advantage of getting this additional information with a single test [19].

### 2.5. PIC cystogram

Rubenstein et al. in 2003 introduced a novel but controversial technique to identify VUR in children presenting with febrile UTIs and negative VCUG [20]. The authors performed the PIC (Positioning the Instillation of Contrast at the ureteral orifice) cystogram by positioning the cystoscope close to the ureteric orifice with the bladder empty and instilling contrast in gravity-aided manner from a height of 1 m using the irrigation port of the scope. The argument that this technique could induce VUR was countered by using a control group of children without UTI and VUR where the PIC cystogram also did not demonstrate reflux. In contrast, all children with febrile UTIs and no VUR on VCUG showed VUR on the PIC cystogram. A multi-institutional study confirmed that PIC VUR could be demonstrated in 82% of children who present with febrile UTI and normal VCUG [21]. The inherent problem with both these studies has been the lack of standardization of the VCUG technique used, absence of upper tract imaging findings, and strict definition of UTI. This latter objection has been addressed in a more recent study by Tareen et al. who evaluated 5 children with recurrent febrile UTI and upper tract changes on DMSA/CT scan [7]. All 5 patients showed VUR on PIC cystography and went on to endoscopic or open VUR correction. The results of these studies indicate that the majority of children with febrile UTI and no VUR on a VCUG would have VUR on a PIC cystogram. Before adding this investigation as a routine modality for children with febrile UTI and negative VCUGs, further prospective randomized studies are indicated to define the population who would benefit from this intervention.

## 3. INDICATIONS FOR IMAGING FOR SUSPECTED VUR

The primary indications for evaluating children for VUR are discussed in this section. There has been considerable evolution of our knowledge about VUR management over the last several years. The role of antibiotic prophylaxis in preventing recurrent infections has been challenged along with an increasing awareness of the development of antibiotic resistance [1–3]. This coupled with identification of other stronger predicting factors for recurrent infection like lower urinary tract dysfunction (LUTS) has led to a more conservative approach in identifying and treating VUR [22]. This section suggests recommendations for the evaluation of children with suspected VUR.

### 3.1. Children with urinary tract infection

The American Academy of Pediatrics: Committee on Quality Improvement recommends a VCUG for all children aged between 2 months to 2 years old following the first febrile UTI [23]. The rationale for this practice is based on the traditional view that there is always uncertainty about whether previous infections were missed, high recurrence rates of UTI, high percentage of children with UTI having VUR and that the risk of renal-acquired cortical defects is highest in younger children. In practice, however, this recommendation is often not followed rigidly primarily because of poor documentation of significant bacteriuria and pyuria which puts in doubt the diagnosis of a UTI. For older children, age at presentation, gender, race, type (febrile or non febrile), frequency of UTI, and social factors must be considered before proceeding to a VCUG. Toilet-trained female children with cystitis are primarily evaluated by a full voiding diary and the dysfunctional voiding symptom score (DVSS) rather than a VCUG [24]. In children with recurrent cystitis a uroflowmetry and US are added. However, in the presence of a well-documented episode of PN or recurrent febrile UTI, a VCUG or RNC is warranted along with a US or DMSA scan to assess the upper tracts. All males with a well-documented febrile UTI should undergo a VCUG. Because of the well-documented low incidence of VUR in black children, a VCUG is not indicated for older black children presenting with UTI. The initial VCUG can be performed after the child is afebrile, clinically stable and the urine is sterile [25]. The dose of antibiotic prophylaxis is doubled a day before the test and continued at therapeutic levels for another day following the test.

### 3.2. Sibling VUR

Primary VUR is the commonest heritable disorder of the genitourinary tract and is inherited as a Mendelian dominant with partial expression [26]. Several studies on sibling VUR have identified factors, which can help predict the risk of sibling VUR. Hollowell in an analysis of 1768 siblings showed a mean VUR incidence of 32%, which was 44% in siblings less than 2 years of age as compared to 9% of siblings greater than 6 years [27]. If the sex of the sibling or proband is considered separately there is no statistical association. On the other hand, female siblings of the female index patient have a higher likelihood of VUR than their male counterparts [28]. Monozygotic twins have an obviously higher risk than dizygotic twins. Hollowell showed that approximately two-thirds of siblings have low grade (I, II) VUR and the spontaneous resolution rate is higher when compared to children diagnosed with VUR after a UTI [27]. Giel et al. presented the long-term outcome of asymptomatic siblings screened for VUR with an initial US [29]. Of the 117 siblings in this study, 11 (9.4%) had abnormal US findings, 5 of which showed VUR on a VCUG. In 85 siblings with an average followup of more than 8 years, none had complications of VUR. Other authors have argued for a more proactive approach in diagnosing VUR in siblings [30, 31]. Houle et al. demonstrated a 26% incidence of cortical defects in siblings and indicated that siblings screened after 2 years of age had a higher risk of renal damage [30]. The alternative argument here is that perhaps these findings represent congenital defects rather than acquired preventable defects.

A tailored approach for siblings therefore could be an RNC or a VUS in siblings younger than the toilet-trained age and US as the initial screening modality for all older siblings. In the presence of any US evidence of cortical damage a VCUG is recommended in children under 5 years of age as they form the subset most at risk of renal damage. Symptomatic siblings at any age are evaluated with a VCUG.

### 3.3. Prenatal hydronephrosis and VUR

VUR is suspected antenatally in the presence of ureteric dilatation and/or hydronephrosis (HN) or following the diagnosis of ectopic kidneys, multicystic dysplastic kidney, and unilateral renal agenesis wherein there is an increased incidence of contra lateral VUR. Van Eerde et al. performed a meta-analysis to review the value of antenatal HN in predicting postnatal VUR [32]. HN was defined as renal pelvic diameter more than 4 mm with or without caliectasis. Among the 1178 cases, the mean prevalence of primary VUR was 14.9%. When stratified by anteroposterior renal pelvic diameter (APD), VUR was diagnosed in 14% of infants with APD ≤ 10 mm and in 12% of infants with APD ≥ 10 mm. It is known that a negative prenatal screening or a normal postnatal US in infants with antenatal HN does not rule out VUR. In a meta-analysis reported by Lee et al., the prevalence of VUR ranged between 4.4% and 14% [33]. There was no correlation between the degree of prenatal HN and the presence or grade of VUR. Similarly, in another study conducted on 108 children with antenatal HN, VUR was detected in 15% and there was no correlation between the degree of pelviectasis on postnatal US and the presence or severity of VUR [34]. Children with antenatal HN and VUR have a more benign course with a higher resolution rate when compared to children diagnosed with VUR following a UTI [35, 36]. Upadhyay et al. followed 25 children with antenatally detected HN and VUR [36]. Reflux was greater than or equal to grade III in 70% of children. VUR resolved in 52% and was downgraded in 24%. Breakthrough urinary tract infection occurred in 4 patients with grades IV and V reflux, and dysfunctional voiding developed in 5. Followup renal scans showed decreased differential function (mean 18%) in 2 units without new scars. A selective approach is advisable for investigating neonates with antenatal HN. If the renal size and parenchyma is unremarkable, it may be reasonable to reserve the VCUG for children with SFU grade 3-4 HN or bilateral HN and in the presence of ureteric dilatation.

### 3.4. Other situations

A routine VCUG is recommended in the work up of children with multicystic dysplastic kidneys (MCDK) based on the reported 15%–25% prevalence of VUR in children with MCDK [37–40]. Miller et al. found a 25% VUR rate in the contralateral kidney in 75 patients with MCDK [37]. In this series, about 50% of children with VUR had grades ≥3, 50% resolved spontaneously by 5 years of age and only 1 of the 75 children required surgical intervention for VUR. Guarino et al. documented that 16% of children with MCDK had VUR and the VUR grade was significantly higher in boys as compared to girls [39]. This finding was also noted in the study by Selzman and Elder, wherein 15% of children showed contralateral VUR, with the prevalence being higher in boys and the white population [40]. Ismaili et al. recommended that 2 successive normal US studies in the neonatal period identifie most significant contralateral anomalies avoiding the use of a routine VCUG [38]. In their study, 61 of the 76 newborns with MCDK had 2 normal neonatal US. Among them, 4 (7%) had low grade VUR which resolved spontaneously in all before 2 years of age. Further studies are needed to validate this finding before stopping routine VCUGs in children with MCDK.

The incidence of VUR in children with unilateral renal agenesis (URA) is slightly higher than MCDK and varies between 24%–28% [39, 41–43]. The VUR can be high grade and shows a lower spontaneous resolution rate as compared to MCDK [41]. Arena et al. evaluated 60 children with renal ectopia (crossed 24, simple 36) [44]. The authors recommended complete urological evaluation of children presenting with renal ectopia. The incidence of associated VUR was 37% with crossed ectopia and 17% with simple ectopia. Unlike MCDK, Guarino et al. noted that girls had a higher grade of VUR and lower resolution rates [39]. In view of the high incidence of neurogenic bladder dysfunction and VUR (20%–47%) children with anorectal malformation should also undergo a VCUG [45].

## 4. WHAT SHOULD THE FIRST INVESTIGATION FOLLOWING PYELONEPHRITIS BE: DMSA OR VCUG?

Primary VUR occurs in less than 1% of the general population but up to 50% of children who present with a UTI will have VUR [46]. Therefore, the detection of VUR is an abnormal finding. The primary reason for identifying VUR as a disease entity has been its association with pyelonephritis (PN), which, if recurrent, can lead to acquired cortical defects and subsequent hypertension and/or end stage renal failure. This perception that the triad of UTI-VUR-nephropathy is an intimate link has driven physicians to actively diagnose and treat VUR over the last 3 decades.

There is a considerable debate regarding the initial investigation following a febrile UTI with several studies highlighting the emerging role of DMSA scan vis a vis the VCUG. The rationale for this argument stems from the recent evidence which has downgraded the importance of VUR as a sole factor in causing long-term renal damage. In fact, our aggressive management of VUR over the last several decades has not impacted long-term renal outcome. Craig et al. reviewed the Australia and New Zealand Dialysis and Transplant Registry between 1971 and 1998 and noted that over the decades, despite a more aggressive identification and treatment of VUR, reflux nephropathy continued to remain a cause of ESRD in about 14% of children registered [47]. This section discusses this current thought process and refocuses our attention to our primary goal which is the identification of risk factors which lead to progressive renal damage.

### 4.1. Pyelonephritis and acquired cortical defects can occur without VUR

Recent studies have demonstrated that acquired renal scarring correlates best with recurrent UTI and not with VUR and primary VUR is neither sufficient nor essential for renal damage. The exception to this rule is secondary reflux associated with bladder outlet obstruction or high-pressure neurogenic bladders. Gordon et al. performed a meta-analysis to determine the value of VUR diagnosis to predict renal damage in children hospitalized with UTI [4]. The analysis evaluated 12 studies comprising 537 children with 1032 kidneys and showed that primary VUR was a poor predictor of renal damage on a DMSA scan in children hospitalized with UTI. A positive VCUG increased the chance of a positive DMSA scan by only about 20% whereas a negative VCUG increased the chance of negative DMSA scan by 8%. The authors concluded that the VCUG could not be used as a primary screening test to detect renal parenchymal damage in children with UTI. Taskinen and Rönnholm noted that fever more than 39°C, CRP > 100 mg/mL, and proteinuria during UTI were predictors of renal damage [48]. The presence of VUR did not increase the risk of renal defects on DMSA scanning. On followup, DMSA scans 2 years following UTI, 9 of the 12 patients who showed evidence of cortical defects did not have associated VUR.

### 4.2. Cortical defects in children with VUR predicts recurrent infection

Mingin et al. retrospectively reviewed records of children who underwent DMSA scans following a febrile UTI or antenatal HN [49]. 88% of the children with an abnormal DMSA scan had grade 3–5 VUR. Of the 51 children with an abnormal DMSA and grade 3–5 VUR 60% had a subsequent breakthrough UTI. In comparison, only 6% of children with similar VUR grade and a normal DMSA scan developed breakthrough infection. Furthermore, only 5% of children with an abnormal DMSA scan showed improvement in VUR grade on followup as compared to a 46% resolution rate in those without DMSA abnormality, a fact only partly attributable to the lower initial grade of VUR in this subset.

### 4.3. A positive DMSA scan identifies significant VUR in most instances

In 303 children less than 2 years of age evaluated with VCUG and DMSA scans after an episode of UTI, Hansson et al. found that 51% had an abnormal DMSA scan and 46% with a positive DMSA scan had no evidence of VUR on VCUG [50]. There was a significant association between ≥grade III VUR and DMSA positive renal lesions. A normal DMSA scan and dilating VUR were found in only 7 children in this study, of which only 1 showed a scarred kidney on followup. None of the 7 children had recurrent UTI on followup. The authors suggested that DMSA could replace VCUG as the primary evaluation for children following a UTI. VCUGs could be selectively performed in children with abnormalities on DMSA scans and this would reduce the number of VCUGs by about half based on the results of this study. The same group conducted a further prospective study to test this hypothesis [51]. In 290 children with UTI in infancy, 52 had VUR which was dilating in 27. An abnormal DMSA scan was documented in 26 of the 27 children with dilating VUR. Tseng et al. also attempted to answer this question whether a normal DMSA can obviate the need for a VCUG following the first UTI [52]. In 142 children, only 5 children with a normal DMSA scan had VUR (all less than or equal to grade 2) and no child with dilating reflux had a normal DMSA scan. Table 1 summarizes these results.

### 4.4. Antibiotic prophylaxis does not prevent recurrent UTI in children with low-grade VUR

The role of VUR, especially lower grades, as a predisposing factor for recurrent UTI is also controversial. Nuutinen and Uhari noted a higher rate of recurrent UTIs in children with grade III–V VUR in comparison with children with grade I-II VUR [53]. It is now believed that the susceptibility for recurrent UTI is more related to a defective urothelial defense mechanism and bladder dysfunction rather than associated VUR. Roussey-Kessler et al. conducted a prospective study on children with grade 1–3 VUR randomized to receive cotrimoxazole or no treatment with UTI on followup as an end point [3]. There was no significant difference in the occurrence of UTI in both groups except in boys with grade 3 VUR (*P* = .04). Garin et al. performed a randomized prospective trial in 218 children with or without VUR who presented with PN, comparing prophylaxis with no prophylaxis [2]. The study only included patients with grade I–III VUR. No statistically significant differences were found among the groups with respect to the rate of recurrent UTI, type of recurrence, rate of subsequent pyelonephritis, and development of renal parenchymal scars. The overall rate of recurrent PN in this study was 5.5% and VUR did not increase the likelihood of PN. The authors concluded that at 1-year followup, grade I–III VUR did not increase the incidence of UTI, PN, or cortical defects. Conway et al. performed a time-to-event analysis on 611 children who are presented with the first UTI to determine the association between antibiotic prophylaxis and recurrent UTI and to identify risk factors for resistance [54]. The factors associated with an increased risk of recurrent UTI in this study were white race, age between 3–5 years, and grade IV-V VUR. Sex and grade I–III VUR were not associated with the risk of recurrence. Moreover, antibiotic prophylaxis was not associated with a decreased risk of recurrent UTI in a multivariable analysis but was a risk factor for antibiotic resistance among children with recurrent UTI.

The problem in interpreting studies attempting to clarify this aspect is the lack of a standardized definition of a febrile UTI and the variability in the methodology of obtaining urine samples. The ongoing randomized intervention for children with vesicoureteric reflux (RIVUR) study is a multicenter, double blinded, randomized, placebo controlled trial which aims to answer the ongoing controversy regarding the role of antibiotic prophylaxis in preventing recurrent febrile/symptomatic UTI in children with VUR diagnosed after a UTI.

In summary:

1. in children presented with a UTI, up to 50% of children may have evidence of upper tract damage without evidence of VUR on a VCUG;
2. the rate of spontaneous resolution of VUR is higher in children with low-grade VUR and a normal DMSA scan;
3. a positive DMSA scan at diagnosis predicts a higher rate of recurrent UTI or breakthrough infections in children with VUR;
4. VUR identification has not altered the ESRD rate related to reflux nephropathy.

The idea behind these studies is to encourage a more selective approach in investigating children who present with a first UTI, contrary to the AAP practice guidelines. A DMSA would be the initial investigation and all children with an abnormal DMSA will then proceed to a VCUG. This would identify the majority of children with dilating/significant VUR who would then benefit from antibiotic prophylaxis, thus reducing both the number of VCUGs and number of children on antibiotic prophylaxis. Such a selective approach is justifiable with one objection being that boys with a potential posterior urethral valve presented outside the neonatal period may be missed with this approach. However, this may be unlikely if a US study is simultaneously performed as part of the routine work up.

### 4.5. MR urography (MRU)

MRU is increasingly being advocated as a single imaging modality, which can be used to provide information obtained on a VCUG and DMSA scan. The primary advantage of the MRU is its ability to distinguish between renal dysplasia (congenital cortical defects) and acquired scarring (acquired cortical defects) [55]. In addition to morphological analysis, MRU can provide information about renal perfusion, concentration, and excretion of contrast media by calculating the renal and calyceal transit times. The Patlak differential function and the calculated Patlak number per mL of renal tissue is considered a surrogate for the single nephron GFR and can therefore serve as an important tool in prognosticating and following children with renal dysplasia.

## 5. FOLLOWUP IMAGING FOR VUR

The ALARA (as low as reasonably achievable) concept has stressed the importance of minimizing radiation exposure in children being followed conservatively after diagnosis of VUR [5]. Thompson et al. devised a theoretical model to study this and conducted a retrospective study in children with primary VUR diagnosed after a UTI to evaluate different strategies of followup and its effect on antibiotic exposure and cost [56]. The authors recommended that children with mild VUR undergo a VCUG every 2 years whereas those with moderate to severe VUR should undergo a VCUG every 3 years. In a survey of the members of the American Association of Pediatrics published in 2001, 99% of the respondents indicated that they would perform a VCUG or RNC every 12–18 months in followup [57]. The current followup protocol should aim to reduce the number of VCUG/RNC performed while children are on antibiotic prophylaxis basing it on the natural resolution decay curve of VUR. It is accepted that all subsequent followup studies following a VCUG should be an RNC.

### 5.1. Factors identified on imaging which predict VUR resolution

Persistence of VUR is more likely in high-grade VUR, in children with bilateral disease (especially in Grade IV and V) and when reflux is diagnosed in the older child. The value of the VCUG and RNC in predicting VUR resolution has been studied. It has been demonstrated that when VUR occurs at less than 60% of expected bladder capacity and the reflux volume is more than 2% of bladder capacity, the resolution is poor [58, 59]. Knudson et al. on a multivariate analysis stated that bladder volume on initial cystogram of greater than 50% of predicted bladder capacity, age younger than 2 years at diagnosis, and a history of prenatal hydronephrosis were significant factors predicting VUR resolution within 2 years [35].

## 6. CONCLUSION

VUR is a heterogenous disorder, and its diagnosis and management continues to remain one of the most controversial problems in pediatric urology. There is a realization that rather than a disease entity, VUR is a marker of overall urinary tract dysfunction, which may predispose to UTI. The primary goal for the treating physician should continue to remain preservation of renal function and preventing the relatively small percentage of acquired renal defects associated with VUR. There has been a paradigm shift in the earnestness with which the diagnosis of VUR is sought after based on an increasing body of evidence which suggests that acquired renal defects are often not related to VUR and that our current modalities for diagnosing VUR are associated with unacceptable radiation exposure and bladder catheterization. The newer modalities do hold promise but further work is warranted before they can replace the existing well-established techniques.

## Figures and Tables

**Table 1:** Results of studies analyzing concordance between DMSA and VCUG findings.

Study (N)	POS DMSA POS VCUG	POS DMSA NEG VCUG	NEG DMSA POS VCUG	NEG DMSA NEG VCUG
Hansson et al. (303)	53 (17%)	103 (34%)	27 (9%); ≥ III VUR in 7	120 (39%)
Tseng et al. (142)	37 (26%)	64 (45%)	5 (3.5%); ≥ III VUR in 0	36 (25%)
Preda et al. (290)	44 (15%)	105 (36%)	8 (2.7%); ≥ III VUR in 1	133 (46%)
